# Crossing the Pillars of Hercules: Understanding transoceanic migrations of seabirds throughout their breeding range

**DOI:** 10.1002/ece3.5079

**Published:** 2019-04-01

**Authors:** Raül Ramos

**Affiliations:** ^1^ Departament de Biologia Evolutiva, Ecologia i Ciències Ambientals, Facultat de Biologia, Institut de Recerca de la Biodiversitat (IRBio) Universitat de Barcelona Barcelona Spain

**Keywords:** Autumn migration, *Calonectris diomedea*, Mediterranean Sea, migratory passage, phenology, seabird movements, spring migration, Strait of Gibraltar, tracking studies

## Abstract

Variability in long‐distance migration strategies is still poorly understood due to the fact that individuals are often tracked from a single colony/population. Transoceanic migrations of Scopoli's shearwaters (*Calonectris diomedea*) across the Strait of Gibraltar (SoG) have been tracked from several breeding colonies isolatedly, and factors related to the variability in phenological schedules among different populations remain, therefore, not well‐understood. Using light‐level geolocator data, I examined the autumn (postbreeding) and spring (prebreeding) migratory passage dates through SoG of four populations of Scopoli's shearwater spread along the longitudinal breeding range of the species. Additionally, I also estimated the at‐sea activity patterns (from immersion data) during both migratory passages, as well as the body size (from morphometric data) of the individuals of these populations. On average, Scopoli's shearwaters leave the Mediterranean (cross SoG) on 31 October ± 1.8 days on their autumn migrations and return on 03 March ± 1.6 days on their spring migrations. At the population level, there was a clear gradient in the timing of crossing SoG: birds from the westernmost populations (Murcia, SE Spain) were the first ones in leaving the Mediterranean while easternmost breeders (Paximada, Crete) were the last ones. In spring, only birds from the largest breeding population (Zembra, Tunisia) seemed to advance their return and crossed SoG significantly earlier than birds tracked at the remaining populations. In both passages, shearwaters from central and eastern populations spent more time flying than their conspecifics from the western Mediterranean. Scopoli's shearwater populations display a differential phenology and behavior in their migratory passages through SoG. The longitudinal gradient in body size already reported for the species could be an evolutionary response to an obvious trade‐off between sharing common wintering grounds in the Atlantic Ocean and the temporal constraints of restoring physiological condition in those grounds.

**OPEN RESEARCH BADGES:**

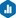

This article has earned an Open Data Badge for making publicly available the digitally‐shareable data necessary to reproduce the reported results. The data is available at https://hdl.handle.net/2445/128784.

## 1 INTRODUCTION

**Figure 1:**
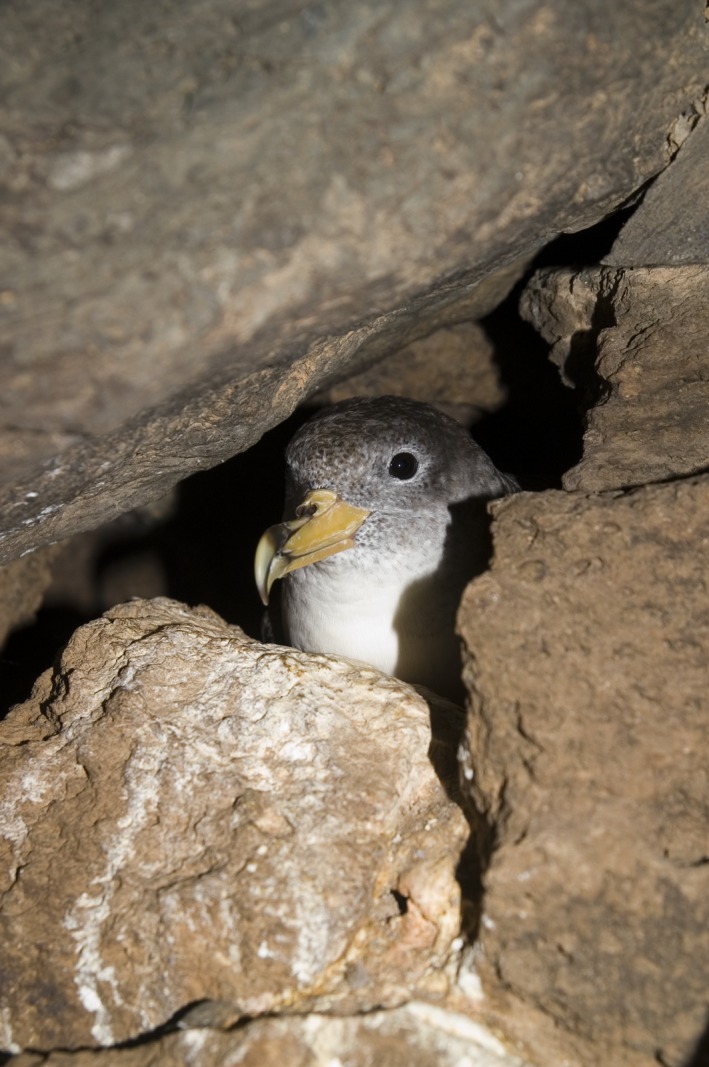
A Scopoli's shearwater (*Calonectris diomedea*) sampled in this study inside its burrow in the colony of Zembra Island, Tunisia. The population size of this colony is the largest throughout the Mediterranean Basin, and hosts more than 75% of the world's breeding population of the species with more than 140,000 breeding pairs (Defos du Rau et al., 2015). Photograph by Raül Ramos

Long‐distance migrations attracted the attention of human mind from the beginning of our times, and a deep understanding of migratory phenology of the species still represents a relevant challenge in current animal ecology (Newton, 2008). Evolutionarily, migratory behavior appeared when a given species took profit of inhabiting two spread areas during the most propitious period of each area, and these profits overcame the costs of long‐distance traveling (Alerstam & Hedenström, 1998). Adjusting the timing of this behavior to maximize benefits and minimize costs is inherent to the evolution of migration (Figure 1) . Concerns on global climate change have spurred many studies on animal phenophases, and among others, there is mounting evidence that migratory phenology is being altered in diverse animal species spread over the world (Hurlbert & Liang, 2012; Rubolini, Saino, & Møller, 2010). Nevertheless, there is a poor understanding on the geographic variability in migratory phenology across the ranges of the species, in spite of its potential relevance for population dynamics and conservation (Martin et al., 2007; Møller, Rubolini, & Lehikoinen, 2008).

**Figure 2:**
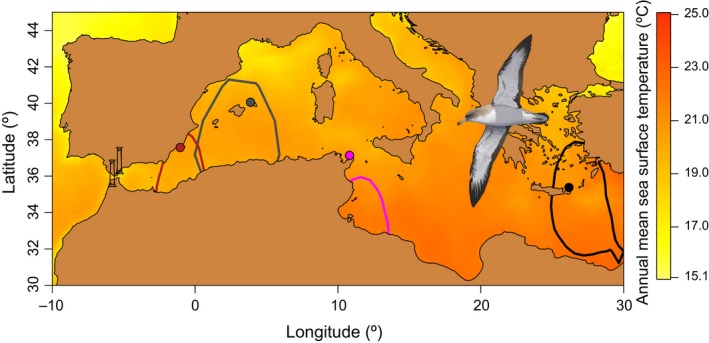
Breeding colonies of Scopoli's shearwater included in the study are shown in colored circles (in brown, gray, purple, and black for Murcia, Balearics, Tunisia and Crete, respectively). All colonies were sampled for 2014–2015 and 2015–2016 migratory annual cycles. Additionally, specific kernel density distributions (50% UDs) calculated for the entire breeding period (from April to September) are depicted for each of the colonies in colored lines (in their respective colors). In the background, I represented the sea surface temperature (ºC) estimated along the Mediterranean Sea throughout the year (average values for 2014 and 2015). The Strait of Gibraltar (36.14° N, 5.35° W; SoG) is depicted with two columns representing the Pillars of Hercules as it was referred in Antiquity (the Rock of Gibraltar: 36.12° N, −5.34° W, and the Monte Hacho: 35.90° N, −5.28° W). The bird silhouette is courtesy of Martí Franch

The Strait of Gibraltar (SoG hereafter; 36.14°N, 5.35°W; Figure 2) is the narrow and shallow oceanographic connection between the Mediterranean Sea and the Atlantic Ocean. It represents a migration bottleneck for most migratory land birds moving between the European and African continents (Miller, Onrubia, Mart, & Kaltenecker, 2016), but also for those diverse marine species moving between the Mediterranean and Atlantic water masses (Abascal, Medina, Serna, Godoy, & Aranda, 2016; Gauffier et al., 2018; Mateos‐Rodríguez & Bruderer, 2012). From last century, many migratory studies used SoG as observatory point for studying spring and autumn passages of diverse marine species because individuals concentrate in a relatively small area for short periods of time, making them visible and countable on board, using radars or even using telescopes from the coast (Garcia, 1971; Hashmi & Fliege, 1994; Silvani, Gazo, & Aguilar, 1999; Stephanis et al., 2008). For instance, thousands of Scopoli's shearwaters (*Calonectris diomedea*), a seabird species endemic of the Mediterranean Sea, concentrate in SoG in specific periods along their annual cycle, and precise phenological monitoring of its migratory passages has been undertaken in the past through direct counts from the coast (Table 1). However, this counting method cannot study individual or population variability in phenology. More recently, technological advances in tracking devices have allowed obtaining individual data on migratory passages for this species (Table 1). However, to date, no study has considered the population of origin along the breeding range of the species as a relevant factor potentially influencing the migration timing of the individuals through SoG.

**Table 1:** A selection of migratory studies of Scopoli's shearwaters (*Calonectris diomedea*) describing phenology of autumn (postbreeding) and spring (prebreeding) passages through the Strait of Gibraltar (SoG). Juvenile and adult estimates are shown separately as it is previously suggested that they might differ (Tellería, 1980)

Breeding population	Age population	Method	*n*	Autumn passage	Spring passage	References
Observational studies	Unknown	In situ		01 November	10 March	Garcia (1971)
	Unknown	In situ		23 October − 10 November	–	Tellería (1980)
	Unknown	In situ		24 October − 10 November 2005	–	Navarrete (2013)
	Unknown	In situ		21 October − 11 November 2006	–	Navarrete (2013)
	Unknown	In situ		26 October − 10 November 2007	–	Navarrete (2013)
	Unknown	In situ		22 October − 09 November 2008	–	Navarrete (2009)
	Unknown	In situ		18 October − 04 November 2010	–	López‐Rodríguez (2011)
Tracking studies						
French colonies	Juveniles	Solar PTT	12	31 October (18 October − 08 November)	–	Péron and Grémillet (2013)
French colonies	Adults	Solar PTT	12	28 October	–	Péron and Grémillet (2013)
Linosa Island	Adults	GLS	60	21 October − 22 November	–	Müller, Massa, Phillips, and Dell'Omo (2014)
Malta	Juveniles	Solar PTT	1	13 November	–	Raine, Borg, and Raine (2011)
Ionian Sea	Adults	GLS	3	03–07 November	18 February − 09 March	Karris (2011)
Crete	Adults	Solar PTT	4	18 October − 08 November	–	Ristow et al. (2000)

The Scopoli's shearwater is a well‐studied seabird that carries out a long and rapid transoceanic migration from its Mediterranean breeding grounds to the wintering areas in the central and south Atlantic Ocean (González‐Solís, Croxall, Oro, & Ruiz, 2007; Ristow, Berthold, Hashmi, & Querner, 2000). All Scopoli's shearwaters traverse SoG twice a year (Paterson, 1987; Tellería, 1980): autumn passage to the Atlantic Ocean takes place between mid‐October and the end of November, and inward movements to the Mediterranean take place between late February and early April (Table 1). Interestingly, the species shows a slight sexual dimorphism in body size, females being generally smaller than males (Thibault et al., 1997), as well as a longitudinal cline in morphometric measurements and body size, with individuals from western populations being larger than eastern ones (Gómez‐Díaz & González‐Solís, 2007; Massa & Lo Valvo, 1986). However, again, no attempt at understanding migratory phenology in relation to sex or population of origin has been conducted for the species.

In this study, I used miniaturized geolocators deployed on birds to evaluate (a) the effect of breeding population and sex of the individuals on the autumn (postbreeding) and spring (prebreeding) migratory passage dates through SoG (gathering data from four populations spread along the longitudinal breeding range of the species; Figure 2). Additionally, I estimated (b) the at‐sea activity patterns of these birds during both migratory passages, and tested the effect of breeding population and daylight (i.e., day vs. night time) on such activity patterns. By these means, I assessed accurate timing of migratory passages and flight activity for each studied population and discussed its relationship with a plausible evolutionary scenario for the morphologic structure described across the species distribution (Massa & Lo Valvo, 1986). From what was known from previous studies, I would expected that different populations of adult Scopoli's shearwaters concentrate around SoG, still in the Mediterranean, and cross it massively and within a few days during their autumn passage (González‐Solís et al., 2007; Tellería, 1980). Similarly, shearwaters could concentrate in common stop‐over sites in the North Atlantic Ocean, before entering synchronously in the Mediterranean during their spring passage (González‐Solís et al., 2007). In both cases, I would expect a decrease in the flight activity of shearwaters in both migratory passages across SoG that allowed feeding, resting and recovering body condition to prepare their last migratory journeys.

## 2 METHODS

### 2.1 Studied species

The Scopoli's shearwater is a medium‐sized Procellariiform species that has a long lifespan of over 30 years (Ramos et al., 2012). The species is a colonial pelagic petrel, highly monogamous, with strong phylopatry and interannual breeding burrow fidelity, and it breeds annually in remote islets and islands (Thibault et al., 1997). Adult Scopoli's shearwaters arrive at breeding colonies in late February or early March, females lay a single egg at the end of May, both mates share incubation duties and chicks hatch in mid‐late July and fledge in late October (Thibault et al., 1997). Both juvenile and adult Scopoli's shearwaters leave the Mediterranean at a similar time (Péron & Grémillet, 2013), although it was previously suggested that juveniles could migrate *ca*. 10 days later in time than adults (Tellería, 1980). It is assumed that all individuals of the species migrate annually toward common wintering areas in the central and south Atlantic (González‐Solís et al., 2007; Ristow et al., 2000), although a very few birds could overwinter in the Mediterranean (Borg, Bonaccorsi, & Thibault, 1999).

### 2.2 Morphometric and complimentary data

Complimentarily to device deployments (detailed below), birds were first sampled for specific morphometric measurements and weight at each breeding site (Table S1). To evaluate differential body size among the colonies and sexes, I first performed a Principal Component Analysis (PCA) using “prcomp” function from R (R Development Core Team, 2017), with culmen, bill depth at nostril, tarsus, and wing length as variables to obtain an estimate of size for every individual bird (i.e., the first principal component [PC1] of the PCA). Secondly, I evaluated the effect of population of origin and sex (as well as their interaction) on the size of the individuals by building a set of competing linear models (LMs) also in R (Table S2). All birds were sexed molecularly from blood samples (diagnostic kit from Durviz, Valencia, Spain).

### 2.3 Sampling design, and tracking and activity data

The present study was conducted at four breeding colonies of the species spread throughout the Mediterranean Sea (Table 2 and Figure 2). In 2014 and 2015, at each colony, geolocator‐immersion loggers (Intigeo‐C250 from Migrate Technology Ltd, Cambridge, UK) were leg‐mounted on breeding adults incubating an egg or rearing a chick. The weight of these geolocators (2.6 g) corresponded to 0.3%–0.4% of bird body mass, thus well below the threshold of 3% above which deleterious effects are more likely to occur (Phillips, Xavier, & Croxall, 2003). One or two years later, geolocators were recovered at each colony place, and I downloaded useful (light and immersion) data from a total of 80 loggers (Table S3).

**Table 2:** Geographical characteristics of sampled populations of Scopoli's shearwaters included in this study. Minimum estimated breeding pairs of each population is also considered

Marine area	Breeding population	Sampled colony	Longitude (°)	Latitude (°)	Estimated breeding population (in pairs)	Reference
Alborán Sea (Western Mediterranean)	Murcia islands	Isla de las Palomas	−1.65	37.35	67–123	Arcos et al. (2009)
Balearic Sea (Western Mediterranean)	Balearic Islands	Cala Morell (Minorca)	3.88	40.05	1,801–6,946	Arcos et al. (2009)
Tyrrhenian Sea (Central Mediterranean)	Tunisia	Zembra Island	10.81	37.17	113,720–176,750	Defos du Rau et al. (2015)
Aegean Sea (Eastern Mediterranean)	Crete	Paximada Islet	26.17	35.38	1,245–2,010	Ristow, Scharlau, Feldmann, Wink, & Wink, (1991)

Intigeo‐C250 geolocators measured light intensity every minute recording the maximum at 5 min intervals. Twilight events from raw light intensities were visually supervised and computed with Intiproc software from Migrate Technology Ltd (Fox, 2015). The sunrise and sunset times were estimated applying the light threshold value of 2. To estimate the sun elevation angle, I calibrated the loggers before deployment and after recovery on an open site without shade. The value of the sun elevation angle was determined individually for every geolocator with at‐colony calibration data, ranging from −2.1 to −4.4. Light‐level data were converted into latitude derived from day length and longitude derived from the time of local midday with respect to Greenwich Mean Time, using Intiproc software (Fox, 2015). This process results in the estimation of two locations every day with a relatively low spatial accuracy (Phillips, Silk, Croxall, Afanasyev, & Briggs, 2004); I removed unrealistic positions that (a) resulted from long periods spent in burrows during incubation; (b) were obtained from light curves showing interference at dawn or dusk (visual inspection); (c) were within the 20 days closest to the equinoxes; or (d) resulted in unrealistic flight speeds (>40 km/h sustained over 48 hr), using iterative backward/forward speed filtering routines (McConnell, Chambers, & Fedak, 1992) written in R (R Development Core Team, 2017).

To evaluate the spatial distribution of the sampled populations across the Mediterranean Sea during summer time, I first estimated core areas used by colonies during the breeding (from April to September) and mapped the 50% Utilization Distributions (UDs; Figure 2) derived from kernel analysis (Calenge, 2006; Phillips et al., 2004).

**Figure 3:**
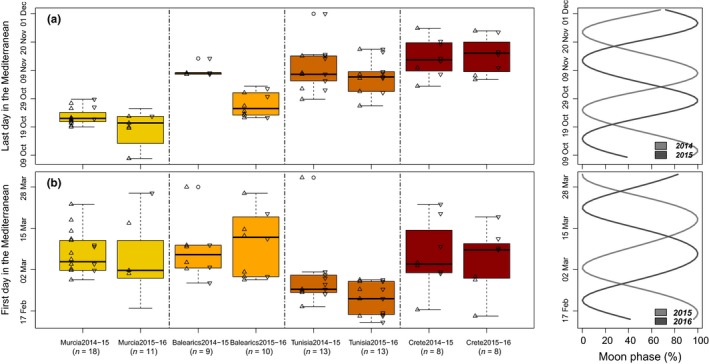
Migratory passages across SoG during autumn and spring migrations of Scopoli's shearwaters. Boxplots represented the (a) last and (b) first dates in the Mediterranean Sea (i.e., autumn and spring migratory passages, respectively) for each sampled colony and annual cycle (each shown separately). Additionally, male and female values are shown over each boxplot, with triangles pointing up and down, respectively. Finally, moonlight (0 represents a new moon, and 100 a full moon) along the passage periods is also depicted on the right panels, separately for each annual cycle

From individualized year‐round tracks, I estimated four key migratory dates: (a) departure date from breeding site, (b) last date in the Mediterranean during their autumn (postbreeding) migration period, (c) first date in the Mediterranean during their spring (prebreeding) migration period, and (d) arrival date at breeding site (Figure 3 and Table S4). Additionally, I also estimated (e) the minimum at‐sea distance between breeding and nonbreeding ground for every track. All dates were first estimated using self‐designed routines written in R and then confirmed by visual inspection on the reconstructed tracks. After checking the normality of the distributions of last and first date in the Mediterranean (split by population), I evaluated the effect of population of origin on the aforementioned variables by fitting a set of candidate Linear Mixed Models (LMMs) and using functions provided by the R packages *lme4* (Bates et al., 2017) and *MuMIn* (Bartoń, 2017). I built a set of competing models, considering population and sex as fixed factors, the effect of moonlight (from 0 during a new moon, to 100 during a full moon) as a potential covariate, and annual cycle and bird identity as random effects (Table 3). The full model included all factors and the interaction between population and sex. Gaussian distribution of error terms and an identity‐link function were used in this modeling. Model selection was based on the Akaike Information Criterion corrected for small sample size (Burnham & Anderson, 1998). When ∆AICc between the best‐supported models was lower than 2 (Johnson & Omland, 2004), I performed model averaging using the function *model.avg* (Bartoń, 2017) of those models to obtain statistical estimates for the variables.

**Table 3:** Linear mixed models (LMMs) testing for the effect of breeding population, sex, and moon phase on last and first dates in the Mediterranean Sea for Scopoli's shearwaters. (a) The structure of candidate models evaluated to explain our data and the values of Akaike's Information Criterion adjusted for small sample sizes (AICc). The most parsimonious models (with ∆AICc < 2.0) are shown in bold. (b) The results of the estimates (±*SE*) obtained from model averaging between the best‐supported models. (c) Proportion of relative variance importance of the fixed effects obtained from model averaging. (d) Estimated variance (±*SD*) of random effects of best‐supported models. All evaluated models included annual cycle (i.e., 2014–2015 and 2015–2016) and individual as random effects

	Scopoli's shearwater crossing the Strait of Gibraltar (SoG)							
	Last day in the Mediterranean					First day in the Mediterranean		
	k	AICc	ΔAICc	AICc Weight		AICc	ΔAICc	AICc Weight
(a) Fixed factors structure (AICc)								
Population*Sex + Moon	12	548.7	7.7	0.008		571.3	3.7	0.064
Population*Sex	11	548.7	7.7	0.008		**568.7**	**1.1**	**0.240**
Population + Sex + Moon	9	**541.1**	**0.1**	**0.355**		572.4	4.8	0.038
Population + Sex	8	**541.0**	**0.0**	**0.372**		569.9	2.3	0.131
Population + Moon	8	**542.9**	**1.9**	**0.141**		570.0	2.4	0.122
Population	7	543.3	2.3	0.117		**567.6**	**0.0**	**0.404**
Sex + Moon	6	598.1	57.1	0.000		584.8	17.2	0.000
Sex	5	597.0	56.0	0.000		582.6	15.0	0.000
Moon	5	605.7	64.7	0.000		583.0	15.4	0.000
Constant	4	604.9	63.9	0.000		580.9	13.3	0.001
(b) Relative variable importance								
Population			1.00		Population		1.00	
Sex			0.84		Sex		0.37	
Moon			0.57		Population:Sex		0.37	
(c) Fixed effect (estimate ± *SE*)								
Murcia (& Males)			17 Oct ± 2.0		Murcia (& Males)		07 Mar ± 3.1	
Balearics (& Males)			30 Oct ± 2.4		Balearics (& Males)		08 Mar ± 2.3	
Tunisia (& Males)			04 Nov ± 2.1		Tunisia (& Males)		24 Feb ± 3.2	
Crete (& Males)			10 Nov ± 2.4		Crete (& Males)		01 Mar ± 5.9	
Females			2.8 ± 1.9		Females		−1.2 ± 2.9	
Moon			2.0 ± 2.4		Murcia:Females		1.8 ± 4.3	
					Tunisia:Females		0.1 ± 3.3	
					Crete:Females		6.4 ± 9.1	
d) Random effects (variance ± *SD*)								
Annual cycle			4.7 ± 2.2		Annual cycle		1.1 ± 1.1	
Individual			17.4 ± 4.2		Individual		31.2 ± 5.6	
Residual			19.4 ± 4.4		Residual		42.4 ± 6.5	

**Figure 4:**
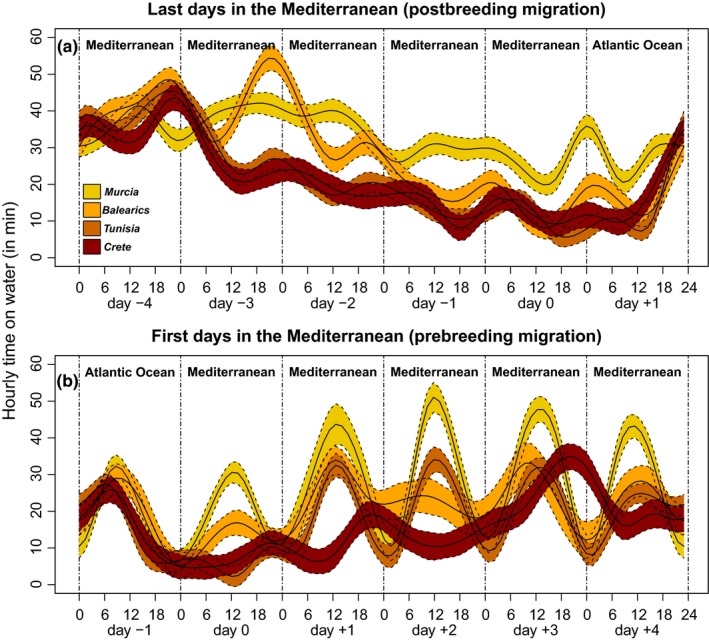
Circadian resting activity throughout both migratory passages across SoG during (a) autumn and (b) spring migrations of Scopoli's shearwaters. Up to four days prior crossing SoG were selected in both cases. The solid lines correspond to the mean of the hourly time (in min) spent resting on the water for each studied population, estimated using generalized additive mixed models (GAMM), and the colored regions around the means delimited by dashed lines represent the associated 95% CI of the slopes. Different populations of Scopoli's shearwaters are plotted as Murcia islands in yellow, Balearic Islands in light orange, Tunisia in dark orange, and Crete in red. As reference, “day 0” corresponds to the (a) last and (b) first date in the Mediterranean Sea, and accordingly, water mass inhabited in each day are shown at the top of each plot (i.e., either Mediterranean or Atlantic). Dashed vertical lines limit the six considered days

The loggers also tested for immersion in sea water every 3 s using 2 electrodes, and provided a value (0–200) corresponding to the sum of positive tests in each 10‐min period, which can be transformed to the proportion of time spent wet, that is, when the bird was sitting on the sea surface or diving. I combined light and immersion data to estimate circadian activity budgets during the crossing of SoG as the resting time on water throughout the day before, during and after the crossing, separately for daylight and darkness periods. I modeled the dynamics of time spent on the water throughout these three days in the four sampled populations using generalized linear mixed models (GLMMs) with binomial error structure and identity‐link function (Table 4). Specifically, I evaluated the associations between population and daylight (*i.e*., day or night) factors with the time spent resting on the water, while accounting for annual cycle and bird identity as random effects. As previously explained, the best‐supported models were selected on the basis of AICc values and their corresponding AICc weights (Johnson & Omland, 2004). For plotting purposes, activity budgets were also modeled using generalized additive mixed models (GAMMs). I used the library *mgcv *in R, based on penalized regression splines and generalized cross‐validation to select the appropriate smoothing parameters (Wood & Augustin, 2002). Generalized additive mixed models combine the utilities of LMMs (Pinheiro & Bates, 2000) and generalized additive models (Hastie & Tibshirani, 1990) so that random factors, fixed factors and nonlinear predictor variables can all be estimated in the same statistical model. Specifically, I included sampled population as a fixed factor, timing throughout the day before, during, and after the passage of SoG as a smooth term, and annual cycle and bird identity as random terms (Figure 4). This allowed me to determine behavioral patterns throughout the autumn and spring passage of SoG in relation to the breeding population of the individuals.

**Table 4:** Generalized linear mixed models (GLMMs) testing for potential effects on daily activity budget of Scopoli's shearwaters (estimated as time spent on the water, in hours) of breeding population and daylight (i.e., day or night) factors along the three days involved in the crossing of SoG (the day before, during and after the crossing). (a) Candidate models evaluated to fit the data corresponding to activity budgets and their associated measures of information (AICc: corrected Akaike's Information Criterion; ΔAICc: AICc increments and AICc Weights). The best‐supported models are shown in bold. (b) Parameter estimates (±*SE*) from the best‐supported models. (c) Estimated variance (±*SD*) of random effects of best‐supported models. All evaluated models included annual cycle and individual as random effects

	*k*	Time on the water_postmigration_ (in hours)			Time on the water_premigration_ (in hours)		
		AICc	ΔAICc	AICc Weight	AICc	ΔAICc	AICc Weight
(a) Fixed factors structure (AICc)							
Population*Daylight	**11**	**55,205.0**	**0.0**	**0.974**	**43,536.6**	**0.00**	**1.00**
Population + Daylight	8	55,212.2	7.2	0.026	43,613.5	76.9	0.00
Population	7	55,316.7	111.7	0.00	43,716.2	179.6	0.00
Daylight	5	55,269.3	64.3	0.00	43,627.6	91.0	0.00
Constant	4	55,373.5	168.5	0.00	43,730.3	193.7	0.00
(b) Fixed effect (estimate ± *SE*)							
Murcia (& Day)			24.8 ± 1.2			26.3 ± 1.3	
Balearics			13.4 ± 1.4			23.4 ± 1.4	
Tunisia			10.7 ± 1.4			14.9 ± 1.4	
Crete			12.8 ± 1.8			11.1 ± 1.8	
Night			5.6 ± 1.2			−9.3 ± 1.2	
Murcia:Night			2.5 ± 1.5			−3.2 ± 1.7	
Tunisia:Night			−1.4 ± 1.5			6.2 ± 1.6	
Crete:Night			−2.6 ± 1.7			11.0 ± 1.8	
(c) Random effects (variance ± *SD*)							
Annual cycle			0.1 ± 0.1			0.1 ± 0.1	
Individual			20.2 ± 4.5			20.0 ± 4.5	
Residual			427.9 ± 20.7			398.6 ± 20.0	

## 3 RESULTS

### 3.1 Morphometrics and size

Scopoli's shearwaters displayed slight differences in most body measurements among the four sampled colonies as well as between sexes (Table S1). The PC1 of the PCA was rather explicative and accounted for 68.8% of the original variability of the four measurements I considered. It was strongly correlated with all body measurements (minimum correlation of −0.73), thus, it well‐estimated the general body size of every individual. AICc values reflected relevant differences in body size among populations and between sexes. Even though measurements were taken by different researchers, body size showed an obvious longitudinal gradient, with individuals being larger and heavier in the western populations and smaller and lighter in the eastern ones (Table S2). In addition, males were generally larger than females in all sampled colonies.

### 3.2 Timing of crossing the Strait of Gibraltar (SoG)

From the geolocation data, I obtained 90 autumn migrations and 84 spring migrations (as a few loggers stopped recording data somewhen during the nonbreeding season) from a total 60 individual Scopoli's shearwaters (21 individuals for Murcia, 18 for Balearics, 13 for Tunisia, and 8 for Crete; Table S3).

At specific level, the Scopoli's shearwater crosses SoG on 31 October ± 1.8 days on its autumn migration (average estimate ± *SE* from the constant model of the LMM modeling described in Table 3), and on 03 March ± 1.6 days on its spring migration (Figure 3). At the population level, there was substantial variation in the dates of passage through SoG in both migrations. Based on AIC values, the best‐supported models always included population of origin and sex as relevant factors, although population had a higher impact in both autumn and spring passages (Table 3). Moon phase was slightly relevant only in the autumn passage modeling, although its effect is partial and not obvious. As random effects, individual identity accounted for a relevant proportion of the total variability in both cases, while annual cycle (i.e., year effect) only contributed with a small proportion of this variability. In the outward migration, there was a clear gradient in the timing of leaving the Mediterranean when one considered the longitude of the population of origin (Figure 3): individuals from Murcia, the westernmost sampled population, were the first birds in leaving the Mediterranean while those easternmost breeders, that is, those from Crete, were the last ones in leaving this Sea, and indeed, no individual from these two extreme populations seemed to co‐occur in that area at the same time. In the return migration, only birds from Tunisia seemed to advance their return and entered the Mediterranean significantly earlier than any other sampled population. Interestingly, in both migratory passages, females tended to cross SoG a few days later than males did, being this effect particularly obvious in those individuals from Crete (Figure 3 and Table 3). Finally, both population and sexual patterns seemed to be consistent over the two annual cycles I considered (Figure 3).

### 3.3 At‐sea activity during migratory passages

Analysis of at‐sea activity patterns during both migratory passages revealed heterogeneity among populations, and daylight and darkness periods (Table 4). In general, individuals from Murcia spent more time resting on the water than any other population during both passages. In addition, birds from Murcia spent more time resting on the water during night time in their outward migration, and more time resting on the water during daylight in their return migration (Figure 4 and Table 4). Interestingly, in return migrations, the time resting on the water during daylight decreased with the longitude of the population of origin, while this time remained similarly low for all populations during night time (Figure 4). Finally, neither annual cycle nor individual identity were relevant contributors to the total variability of random effects.

## 4 DISCUSSION

### 4.1 Migratory ecology of the Scopoli's shearwater

As expected, all Scopoli's shearwaters tracked in this study left the Mediterranean Sea after breeding. In general, phenological estimates derived from this study matched well previous records of both autumn (postbreeding) and spring (prebreeding) passages through SoG (Table 1). However, this data also revealed an unexpected longitudinal gradient in the timing birds crossed SoG, with birds from western populations leaving the Mediterranean much earlier than birds from eastern populations. In addition, significant differences among populations (i.e., several weeks of difference) were consistent over the two annual cycles I analyzed, suggesting that this behavioral pattern occurs regularly across years. This longitudinal pattern results in part from the fact that individuals of different populations already started autumn migrations at different timing within the annual schedule (see Table S4 in Appendix), and this pattern is further amplified by the fact that those populations breeding further east from SoG spent more time crossing the Mediterranean. However, the reverse trend was not true for spring migrations, and birds from the eastern Mediterranean were not the first ones in entering the Mediterranean. Only birds from Tunisia returned consistently earlier (over the two annual cycles) and arrived earlier to the breeding grounds. Such unexpected pattern was likely related to the size of the sampled colony (i.e., Zembra Island), the largest in the Mediterranean, that hosts more than 75% of the world's breeding population of the species (Defos du Rau et al., 2015), concentrated in an island of less than 450 ha. Thus, in Zembra, competition for finding a breeding site within the colony may be stronger than in other colonies and individuals may have a greater pressure to migrate earlier in their prebreeding movements than individuals from any other colony (Sparks et al., 2005). A similar reasoning could be argued for the finding that males cross SoG earlier in the breeding season than females; they tended to leave the Mediterranean earlier than females, and also, enter back earlier, although in both cases, statistical evidence was not robust. As explained before, Scopoli's shearwaters display a sexual dimorphism in bill and body sizes (Granadeiro, 1993; Massa & Lo Valvo, 1986), which is assumed to confer an advantage to males for physical combats against conspecifics in nest defense during the prelaying period (Granadeiro, Burns, & Furness, 1998; Navarro, Kaliontzopoulou, & González‐Solís, 2009). Thus, the earlier migration of males and arrival at the breeding grounds likely reflect the competition for nesting sites (Granadeiro et al., 1998).

At‐sea activity patterns of the four study populations also differed considerably among themselves throughout both migratory passages. In autumn migrations, before crossing SoG, shearwaters from central and eastern populations spent higher proportion of time flying than their conspecifics from the western Mediterranean (Figure 4a). Similarly, in the spring migration, both central and eastern populations also spent higher proportion of time flying during the first days in the Mediterranean Sea, particularly during daylight. From these results, one can assume that eastern populations leave and enter the Mediterranean, flying directly toward SoG, in their respective autumn and spring migrations, without much resting on their way. This differential flying behavior and effort during migration could simply reflect the distance between SoG and their own breeding sites, which is much larger for those eastern populations of Scopoli's shearwaters.

### 4.2 Evolutionary consequences of remote breeding

An important consequence of the temporal gradient defined in the autumn passage through SoG is that, as all populations of Scopoli's shearwaters shared common nonbreeding grounds and migrated similar distances on their autumn and spring movements (Table S4; V. Morera‐Pujol, C. Péron, P. Catry, M. Magalhães, J. M. Reyes‐González, J. P. Granadeiro, T. Militão, M. P. Dias, D. Oro, G. Dell'Omo, M. Muller, V. H. Paiva, B. Metzger, V. Neves, J. Navarro, G. Karris, S. Xirouchakis, J. G. Cecere, A. Zamora‐López, M. G. Forero, F. De Felipe, Z. Zajková, M. Cruz‐Flores, D. Grémillet, J. González‐Solís, R. Ramos in prep), individuals from eastern populations of the species spent less time in the wintering grounds. It is well‐known that long‐distance migrants use remote and productive wintering areas during nonbreeding periods for recovering body condition, for intense feather replacement (i.e., molt), and for fat accumulation in better environmental conditions than in their own breeding grounds (Alerstam, Hedenström, & Susanne, 2003). Therefore, easternmost breeding populations of Scopoli's shearwater would have less time for their self‐maintenance duties in such remote areas than their conspecifics from the western Mediterranean populations. In this regard, the longitudinal gradient in body size I reported here (also described elsewhere), and the smaller size of birds breeding in those easternmost populations could be explained by the fact of having less time available for recovering from breeding and migrating in the common nonbreeding grounds of the Atlantic Ocean. Indeed, smaller species need less energy reserves and can recover faster from fasting periods than larger species do (Gardner, Peters, Kearney, Joseph, & Heinsohn, 2011; Millar & Hickling, 1990), and this reasoning can be applied intraspecifically. In several examples of migratory species, increasing body size is predicted to increase time and energy cost of migration (Hamilton, 1961; Hedenström, 2008), and very often, the migration distances covered by the species are inversely related to their body sizes (Hedenström, 2006). In the case of Scopoli's shearwaters, the differential length of the period spent in the winter quarters, where the restoration of body condition occurs, could have conditioned differentially the body size of their populations.

Classically, this morphometric gradient within the Mediterranean Sea has been interpreted as a transition cline between the larger and sibling species from the Atlantic Ocean, the Cory's shearwater (*Calonectris borealis*), and the smaller individuals of Scopoli's shearwater from the Mediterranean (Granadeiro, 1993; Massa & Lo Valvo, 1986). However, the lack of genetic structuring among Mediterranean populations (Genovart et al., 2013; Gómez‐Díaz, González‐Solís, & Peinado, 2009; Zidat et al., 2017) and the absence of the reverse morphometric trend across Cory's shearwater populations in the Atlantic (Granadeiro, 1993) do not support this classical hypothesis of mixture and dilution of large body sizes from east to west inside the Mediterranean basin. Other hypothesis suggested that food web productivity nearby the breeding area of each shearwater population could influence birds' body size (Massa & Lo Valvo, 1986). Indeed, studies based on annual catch estimates of small pelagic fish suggested that central Mediterranean accommodates the largest fish stock, whereas western Mediterranean has smaller stocks, and eastern Mediterranean accounts for the smallest fish stock (Stergiou et al., 2016). I concur with the idea that overall productivity of a given area (measured, for instance, and annual fishery catches) might relate to the size of breeding populations of Scopoli's shearwaters (see estimated breeding populations in Table 2), but not necessarily to the size of their individuals. A alternative climatic hypothesis, known as Bergmann's rule, describes a positive relationship between body size and latitude, smaller individuals being often found at lower latitudes where climate is typically warmer (Bergmann, 1847). In the case of the Mediterranean Sea, specific environmental characteristics were defined across longitude (Coll et al., 2010), and this variability could have promoted divergence in body size among populations of diverse marine species through local adaptations (Zotier, Bretagnolle, & Thibault, 1999). Indeed, warmer waters of the eastern Mediterranean (Figure 2), for instance, could certainly contribute to the late start of autumn migration described here for this population of Scopoli's shearwater (Table S4). However, a climate modulating effect on the morphometric cline described in the species across longitude is not obvious (Gómez‐Díaz & González‐Solís, 2007). Therefore, in addition to such climatic constraints/influences, the present study added complementary evidence that individuals and populations of Scopoli's shearwater breeding in the eastern Mediterranean could be smaller because of their relative shorter wintering periods, which might have constrained their capacity of physiological recovery during the nonbreeding period.

## 5 CONCLUSIONS

Scopoli's shearwater populations displayed a differential migratory passage through SoG: birds originating from the eastern Mediterranean Sea not only migrated later through SoG than western populations, but also spend less time in their common Atlantic wintering grounds. In addition, Scopoli's shearwaters behaved differently during the crossing of SoG according to their breeding origin: birds from eastern populations flew higher proportion of time while crossing SoG, both at leaving and entering the Mediterranean Sea. I hypothesize that the longitudinal gradient in size reported for the species could be an evolutionary response to an obvious trade‐off between sharing remote, common wintering grounds with other populations and the temporal constraints of restoring physiological/body condition in those grounds. This reasoning complemented previous hypotheses which suggested that several environmental determinants conditioned such a morphometric gradient. This study contributed to the idea that smaller body sizes of the Scopoli's shearwaters from the eastern Mediterranean might be a consequence of (a) flying longer distances to reach common wintering areas, (b) optimizing the investment of energy stores for migrating, and (c) facilitating the restoration of body condition in those winter quarters. Therefore, the finding I reported here, and its reasoning, brought new light into the unsolved explanation for the morphologic structure of the Scopoli's shearwater, but also highlighted the potential relevance of accurate phenological schedules defined across the breeding range of any species. Complementary further studies examining the molt and the physiological status of birds of different populations at the time of migration (Ramos, González‐Solís, & Ruiz, 2009) could help ensuring the aforementioned hypothesis.

## CONFLICT OF INTEREST

None declared.

## AUTHOR CONTRIBUTIONS

R.R. conceived the study, analyzed the data, and wrote the paper.

## Data Availability

Data used in this study are deposited in the University of Barcelona (UB) Digital Repository (http://hdl.handle.net/2445/128784). Data are split in three datasets accounting for positioning data in the Mediterranean (GLSdata.E&E.csv), activity/immersion data during autumn and spring migrations (ACTdata.hourly.E&E.csv), and morphological data (Biometry.E&E.csv) of the four sampled population.
