# Supplementary material for: Crossing the Pillars of Hercules: Understanding transoceanic migrations of seabirds throughout their breeding range
Source: Ecol Evol. 2019 Apr 1;9(8):4760–71. doi: 10.1002/ece3.5079 (PMC6476834; doi:10.1002/ece3.5079)
Supplement: Supplementary file 1 [file ECE3-9-4760-s001.docx]

**Table S1.** Six morphometric measurements and weight (mean ± SD) of Scopoli’s shearwaters sampled at the four breeding locations that I included in the study. A general estimate of body size is included in the last column, as the first Principal Component of a PCA computed with culmen, bill depth at nostril, tarsus, and wing length variables (explaining the 68.8% of the morphometric variability).

| Breeding  population | Sampled  colony | Researcher  /Sampler | Sex | n | Culmen | Maximum  bill depth | Bill depth  at nostril | Bill-head | Tarsus | Wing  length | Weight | PC1  (size) |
| --- | --- | --- | --- | --- | --- | --- | --- | --- | --- | --- | --- | --- |
| Murcia islands | Isla Palomas | Elisa Miquel | male | 28 | 52.9 ± 1.7 | 19.8 ± 1.1 | 14.6 ± 1.0 | 110.1 ± 3.2 | 55.7 ± 1.4 | 354.0 ± 8.2 | 672.3 ± 64.8 | 22.0 ± 5.4 |
|  |  |  | female | 10 | 48.1 ± 2.0 | 17.8 ± 0.8 | 12.5 ± 0.5 | 103.9 ± 3.1 | 53.4 ± 2.0 | 344.7 ± 3.7 | 599.4 ± 37.7 | 11.2 ± 3.3 |
| Balearic Islands | Cala Morell | Fernanda de | male | 21 | 52.9 ± 1.8 | 19.6 ± 0.9 | 14.3 ± 0.6 | 109.9 ± 2.0 | 56.0 ± 1.4 | 355.1 ± 10.4 | 662.4 ± 42.9 | 22.2 ± 4.5 |
|  | (Minorca) | Felipe | female | 19 | 49.2 ± 1.3 | 17.9 ± 0.6 | 12.8 ± 0.6 | 102.8 ± 3.9 | 54.3 ± 3.8 | 347.4 ± 6.7 | 573.7 ± 41.5 | 12.8 ± 4.8 |
| Tunisia | Zembra Island | R. Ramos | male | 12 | 52.0 ± 1.1 | 19.6 ± 0.6 | 14.2 ± 0.6 | 109.4 ± 1.2 | 55.4 ± 1.0 | 349.5 ± 5.2 | 663.8 ± 67.0 | 19.3 ± 2.3 |
|  |  |  | female | 23 | 48.9 ± 1.4 | 17.7 ± 1.5 | 12.9 ± 1.1 | 105.3 ± 2.9 | 53.4 ± 1.3 | 347.0 ± 8.1 | 623.3 ± 65.3 | 13.2 ± 4.5 |
| Crete | Paximada Islet | R. Ramos | male | 25 | 50.5 ± 1.4 | 18.7 ± 0.6 | 13.6 ± 0.5 | 106.3 ± 2.0 | 54.5 ± 1.2 | 348.2 ± 7.9 | 595.2 ± 47.7 | 15.8 ± 3.8 |
|  |  |  | female | 33 | 47.7 ± 1.3 | 17.2 ± 0.6 | 12.1 ± 0.5 | 102.1 ± 1.7 | 52.5 ± 1.3 | 341.3 ± 7.0 | 569.5 ± 55.6 | 8.3 ± 3.7 |

**Table S2.** Linear models (LMs) testing for the effect of breeding population and sex on estimates of body size (i.e., that first Principal Component of the PCA) of individual Scopoli’s shearwaters. (a) The structure of candidate models evaluated to explain our data and the values of Akaike’s Information Criterion adjusted for small sample sizes (AICc). The most parsimonious models (with ∆AICc <2.0) are shown in bold. (b) The results of the estimates (± standard error, SE) obtained from model averaging between the best-supported models.

|  |  | Body size (PC1) | | |
| --- | --- | --- | --- | --- |
| *a) Fixed factors structure (AICc)* | k | AICc | ΔAICc | AICc Weight |
| **Population*Sex** | **9** | **992.3** | **0.8** | **0.405** |
| **Population+Sex** | **6** | **991.5** | **0.0** | **0.595** |
| Population | 5 | 1098.8 | 107.3 | 0.000 |
| Sex | 3 | 1031.4 | 39.8 | 0.000 |
| Constant | 2 | 1134.3 | 142.7 | 0.000 |
| *b) Fixed effect (estimate±SE)* |  |  |  |  |
| Murcia (& Male) |  |  | 21.9 ± 0.8 |  |
| Balearics |  |  | 21.6 ± 0.9 |  |
| Tunisia |  |  | 20.2 ± 1.2 |  |
| Crete |  |  | 16.1 ± 0.8 |  |
| Female |  |  | -9.4 ± 1.7 |  |
| Balearics:Female |  |  | 0.6 ± 1.5 |  |
| Tunisia:Female |  |  | 1.9 ± 2.7 |  |
| Crete:Female |  |  | -1.3 ± 2.0 |  |

**Table S3.** Summary of geolocator deployments, recoveries, and profitable data gathered from the four Scopoli’s shearwater colonies I included in the study. Notice that some loggers were recovered two years after their deployment, and therefore provided two years of data.

| Sampled  population | Sampled  colony | Annual cycle | Deployments | Recoveries | Tracks with  profitable data |
| --- | --- | --- | --- | --- | --- |
| Murcia islands | Isla Palomas | 2014-2015 | 24 | 17 | 18 |
|  |  | 2015-2016 | 15 | 12 | 11 |
| Balearic Islands | Cala Morell | 2014-2015 | 16 | 14 | 9 |
|  | (Minorca) | 2015-2016 | 27 | 15 | 10 |
| Tunisia | Zembra Island | 2014-2015 | 25 | - | 13 |
|  |  | 2015-2016 | - | 13 | 13 |
| Crete | Paximada Islet | 2014-2015 | 25 | - | 8 |
|  |  | 2015-2016 | - | 9 | 8 |
| **Total** |  |  | **132** | **80** | **90** |

**Table S4.** Migration characteristics (median ± SE, and sample size and range in parentheses) of the four sampled colonies of Scopoli’s shearwaters. I provided: the date of leaving the colony (breeding site), the last and first dates in the Mediterranean Sea (i.e., autumn and spring passage through SoG), and the date of arrival at the colony place, for each sampled colony and annual cycle. Complementarily, I also included the minimum distance between breeding and non-breeding grounds, computed at sea and for every individual Scopoli’s shearwater.

| Sampled  population | Annual cycle |  | Leaving  the colony | Last day in  the Mediterranean | First day in  the Mediterranean | Arrival at  the colony | Minimum distance between  breeding and non-breeding  grounds (in km) |
| --- | --- | --- | --- | --- | --- | --- | --- |
| Murcia | 2014-2015 |  | 19 Oct ± 0.7  (18; 16 Oct\|27 Oct) | 20 Oct ± 0.6  (18; 17 Oct\|27 Oct) | 02 Mar ± 2.5  (18; 26 Feb\|11 Apr) | 02 Mar ± 2.6  (18; 26 Feb\|12 Apr) | 6347.3 ± 812.9  (18; 2612.7\|12862.9) |
|  | 2015-2016 |  | 20 Oct ± 0.8  (8; 17 Oct\|24 Oct) | 21 Oct ± 0.8  (8; 17 Oct\|24 Oct) | 28 Feb ± 3.1  (7; 17 Feb\|15 Mar) | 29 Feb ± 2.9  (8; 17 Feb\|15 Mar) | 6865.5 ± 904.4  (8; 2653.8\|8212.1) |
| Balearics | 2014-2015 |  | 06 Nov ± 0.5  (9; 05 Nov\|10 Nov) | 06 Nov ± 0.6  (9; 06 Nov\|12 Nov) | 05 Mar ± 2.1  (8; 25 Feb\|16 Mar) | 07 Mar ± 1.8  (9; 01 Mar\|18 Mar) | 4316.0 ± 1002.1  (9; 3093.9\|12862.9) |
|  | 2015-2016 |  | 25 Oct ± 1.5  (12; 17 Oct\|01 Nov) | 25 Oct ± 1.3  (12; 20 Oct\|02 Nov) | 07 Mar ± 2.2  (10; 26 Feb\|17 Mar) | 05 Mar ± 2.0  (12; 27 Feb\|18 Mar) | 8438.0 ± 710.4  (10; 3204.6\|8568.8) |
| Tunisia | 2014-2015 |  | 03 Nov ± 2.1  (13; 26 Oct\|25 Nov) | 06 Nov ± 2.3  (13; 27 Oct\|29 Nov) | 23 Feb ± 1.1  (13; 18 Feb\|04 Mar) | 26 Feb ± 1.1  (13; 20 Feb\|06 Mar) | 5584.2 ± 588.1  (13; 3563.1\|8991.7) |
|  | 2015-2016 |  | 03 Nov ± 1.8  (13; 22 Oct\|13 Nov) | 05 Nov ± 1.8  (13; 25 Oct\|15 Nov) | 20 Feb ± 1.4  (13; 13 Feb\|26 Feb) | 24 Feb ± 1.4  (13; 15 Feb\|29 Feb) | 5842.3 ± 576.4  (13; 3673.8\|8991.7) |
| Crete | 2014-2015 |  | 30 Oct ± 3.1  (8; 11 Oct\|05 Nov) | 11 Nov ± 2.5  (8; 02 Nov\|23 Nov) | 02 Mar ± 3.5  (8; 17 Feb\|18 Mar) | 06 Mar ± 3.8  (8; 25 Feb\|27 Mar) | 5729.6 ± 668.1  (8; 5252.1\|10415.6) |
|  | 2015-2016 |  | 30 Oct ± 2.7  (8; 24 Oct\|18 Nov) | 13 Nov ± 2.4  (8; 04 Nov\|22 Nov) | 07 Mar ± 3.6  (7; 15 Feb\|17 Mar) | 10 Mar ± 3.6  (8; 19 Feb\|20 Mar) | 5618.9 ± 593.0  (8; 5321.7\|10305.8) |
